# Bacterial minicells to the rescue: cyto‐Immunotherapy for the treatment of late stage cancers with minimal to no toxicity

**DOI:** 10.1111/1751-7915.13952

**Published:** 2021-10-19

**Authors:** Himanshu Brahmbhatt, Jennifer A. MacDiarmid

**Affiliations:** ^1^ EnGeneIC Pty Ltd Building 2, 25 Sirius Road, Lane Cove West Sydney NSW 2066 Australia

In the early 1900s, German chemist, Paul Ehrlich commenced developing drugs to treat infectious diseases and coined the term ‘chemotherapy’ defining it as the use of chemicals to treat disease. He developed the first alkylating agents, to treat cancer.

During the past 100 years, the major ‘cancer cure’ concepts evolved from;

- a magic bullet with a single drug able to kill tumour cells,
- a magic bomb combining surgery, radiotherapy and chemotherapy to achieve higher levels of anti‐tumour efficacy,
- monoclonal antibodies to receptors over‐expressed on tumour cell surface to reduce toxic side effects and achieve tumour‐targeted therapy,
- synthetic nanoparticles and polymers to package drugs and deliver them to tumour cells to reduce toxic side effects and achieve higher anti‐tumour efficacy,
- molecularly targeted drugs that targeted specific oncogenic proteins to avoid killing normal cells,
- checkpoint inhibitor immunotherapy to reactivate the patient’s own immune system,
- Chimeric antigen receptors to give T cells the ability to target a tumour‐specific protein and T cell activating function in a single receptor.

While some spectacular cures were observed with each of these approaches, most patients experienced tumour relapse and eventually succumbed to the disease. These advances provided an incremental advance in the treatment of cancer and almost all of them were associated with moderate to severe toxicity. Throughout this time, there was a major effort to discover antigens that were tumour‐specific with a hope that tumour‐targeted therapies could be developed with minimal toxicity to normal tissues. This effort has yet to bear fruit.

So, the question arises as to what are the root causes of this limited success. Some key points to consider are as follows.

1. Most cancers are diagnosed when patients are at Stage III or IV. It is well known that tumour heterogeneity at this stage is extensive and most tumour cells have already elaborated multiple drug resistance and immune‐suppression mechanisms. Cell proliferation assays on tumour cells isolated from biopsies show near complete resistance to most available chemotherapeutic drugs (Sagnella *et al*., 2020). It is not surprising that first, second and third line chemotherapies only provide partial responses followed by tumour recurrence and ultimately death. It is clear that chemotherapy will most likely fail to kill all the tumour cells in a Stage IV cancer patient. This approach generally only achieves partial remission, increase in overall survival by a few weeks to months and very serious toxicity at the end stage of the patient’s life.
2. It is well recognized that the patient’s own immune system, if suitably activated, can also exert anti‐tumour efficacy. Unfortunately, many of these chemotherapies severely damage the immune system preventing it from mounting anti‐tumour responses.
3. Pharmaceutical science and drug development over the past century has been driven by a belief that single molecular entities directed to a single target can cure this disease. For example, salbutamol (Ventolin) could alleviate the symptoms of asthma or chronic obstructive pulmonary disease, penicillin and other antibiotics could cure bacterial infections. The same principle has been applied to cancer therapy for example, when it was discovered that cytotoxic T cells were being suppressed by PD1 ligands secreted by tumour cells, it was thought that anti‐PD1 inhibitor monoclonal antibody would reactivate CD8+ T cells and would effect a cure. While anti‐PD1 inhibitors do help in some cancers, the disease is far too complex with thousands of molecular events in action simultaneously to allow tumours to escape the immune system and aggressively overcome host defences (Bagchi and Yuan, 2021). Since 2011, 7 different checkpoint inhibitors have been approved by the FDA and interestingly over 30 new checkpoint inhibitors are in development and many new ones are being discovered. Each of these have moderate to severe toxic side effects and clinical trials are in progress to try and combine two or more such inhibitors to determine if a higher level of anti‐tumour efficacy can be achieved. For anti‐PD‐1 alone, over 1,500 clinical trials are in progress world‐wide. It is surprising that millions of dollars are being poured into such an approach which is fraught with failure. Similarly, over 300 CAR‐T ‘me‐too’ therapies are in development with more than 500 clinical trials in progress. This ‘herd‐mentality’ is a significant part of the problem where there is a flawed assumption that single molecule/single target will cure cancers.

This issue focuses the mind on taking a step back and learning from history, getting to the roots of the problem and deciphering if there is a better way to address it so that we are able to pursue a more realistic path in the decade to come.

Interestingly, microbial cells may offer solutions to these seemingly insurmountable problems.

Given that most cancer cells elaborate a sophisticated plethora of drug resistance and immune‐suppressive mechanisms, is there any way to overcome multi‐drug resistance in cancer cells? Going after each different drug resistance mechanism or individual targets would again lead to hundreds of drugs with attendant toxicities and appropriate therapy would be impossible. Additionally, experience shows that targeting just one or two pathways can be easily overcome by tumour cells since they elaborate a multitude of different drug resistance pathways which can overcome single hits.

It is known for some time that there are cytotoxic drugs that can overcome multiple drug resistance mechanisms simply by virtue of the fact that these drugs are super‐poisons. Examples include (i) PNU‐159682, a metabolite of the anthracycline nemorubicin, a highly potent DNA topoisomerase I inhibitor which is over 2000‐fold more toxic than conventional drug doxorubicin, (ii) Duocarmycin which is a DNA minor groove‐binding alkylating agent, (iii) Maytansine, a benzoansamacrolide, a highly potent microtubule‐targeted compound that induces mitotic arrest and kills tumour cells at sub‐nanomolar concentrations etc. Unfortunately, these drugs cannot be administered in patients as free chemotherapy since they are too toxic and would kill a person due to rapid and widespread killing of normal cells. These drugs are being developed as antibody‐drug conjugates but even then, they are seriously toxic in patients.

If it were possible to safely administer these drugs into cancer patients so that the drug is specifically taken up inside cancer cells and not normal cells, then it should be possible to kill even the most drug‐resistant cancer cells. Bacterial minicells which are anucleate nanoparticles produced as a result of inactivating the genes that control normal bacterial cell division (de Boer and Crossley, 1989; Lutkenhaus and Addinall, 1997; Ma and King, 2004) thereby derepressing polar sites of cell fission, may provide a solution to these and other obstacles to cytotoxic drug delivery.

Genetically defined *min*CDE‐ chromosomal deletion mutants were generated from *Salmonella enterica* serovar Typhimurium (S. Typhimurium) (MacDiarmid *et al*., 2007, 2009). The minicells were shown to be 400 nm in diameter (hence referred to here as nanocells or EDV™; EnGeneIC Dream Vector), anucleate, non‐living and carry the outer and inner membrane surrounding an empty cytoplasm. The EDVs were shown to readily package a range of different cytotoxic drugs or nucleic acids including the super‐poisons mentioned above and interestingly, once packaged in the cytoplasm, the drug does not leak out of the EDV as was demonstrated in Phase I and Phase IIa clinical trials (on‐going) in over 170 end‐stage cancer patients who have received over 2400 EDV doses carrying different cytotoxic drugs (Kao *et al*., 2015; Solomon *et al*., 2015; van Zandwijk *et al*., 2017; Sagnella *et al*., 2020). These patients show little to no toxicity despite repeat intravenous (i.v.) dosing with many patients receiving 15 to 70 repeat doses. Given that the EDV surface is coated with lipopolysaccharide (LPS), single‐chain bispecific antibodies were attached to the EDV surface where one arm of the antibody is directed to the O‐polysaccharide epitopes and the other arm is directed to a tumour cell surface receptor for example Epidermal growth factor receptor (EGFR) which is found on the surface of over 70% of solid tumours.

Drug‐packaged, antibody‐targeted EDVs can be readily produced in high yield and purified free of parental bacteria, membrane blebs, nucleic acids, cellular debris and free endotoxin, using pharmaceutical cross‐flow and dead‐end filters. The final therapeutic is lyophilized and stored and shipped anywhere in the world at 4°C. The vials are stored in the hospital pharmacy and when a patient is to be dosed, 2 ml of sterile water for injection is added to reconstitute the EDVs.

The EGFR‐targeted, PNU‐packaged EDVs are injected i.v. and because of their relatively large size (~ 400 nm diameter) they are retained in the normal blood circulation since the gaps between endothelial cells lining the blood vessels is less than 2 nm. However, it is known that cancer cells require access to blood vessels for growth and metastasis and hence they over‐express pro‐angiogenic factors which leads to the development of disorganized blood vessel networks that are fundamentally different from normal vasculature. Tumour vasculature is typified by aberrant structural dynamics and vessels that are immature and hyperpermeable (Siemann, 2011). The fenestrations in these blood vessels can range for 20 nm to over 4 μm (Hashizume *et al*., 2000).

The EDVs being 400 nm rapidly fall out of these fenestrations and enter into the tumour microenvironment and since they carry the bispecific antibody on the EDV surface, the anti‐EGFR component binds to EGFR on the tumour cell surface. This provokes macropinocytosis and the EDVs are taken into the early endosomes, followed by lysosomes and broken down in these organelles releasing the drug PNU‐159682. The drug enters into the tumour cell cytoplasm and the nucleus and intercalates with the chromosomal DNA resulting in tumour cell apoptosis. In the event that a tumour type does not express EGFR for example liver cancer, which expresses asialoglycoprotein, then the bispecific antibody can be changed to anti‐asialoglycoprotein while the anti‐O‐polysaccharide component remains constant. Similarly, HER‐2 positive breast cancers can be targeted via anti‐HER2/anti‐O‐polysaccharide bispecific antibody.

This is the first time a super‐cytotoxic drug has been administered into human cancer patients with no toxicity. The double membrane structure of the EDVs prevented leakage of the drug in general circulation, the large size of the EDVs allow it to avoid the normal tissues that are surrounded by normal sealed blood vessels, the tumour‐associated leaky vasculature allows the EDV to enter specifically into the tumour microenvironment, the bispecific antibody targeting of the EDVs allow it to enter specifically into tumour cells and the lysosomal degradation machinery allowed the EDV to be broken down intracellularly and release the drug that could overcome drug resistance and for the first time, kill tumour cells that are highly drug resistant with no toxicity. All 170 cancer patients treated so far were end‐stage palliative care patients who had run out of all treatment options. Highly significant anti‐tumour efficacy has been observed in mesothelioma (Kao *et al*., 2015), glioblastoma and pancreatic cancer (Sagnella *et al*., 2020).

Given that the body’s own immune system has the potential to augment anti‐tumour efficacy, it would be ideal if one could simultaneously harness this potential without toxicity associated with current immunotherapies.

The EDVs that are in general blood circulation, and which have not entered into the tumour microenvironment, are rapidly recognized as foreign via the pathogen‐associated molecular patterns (PAMPS) such as LPS by professional phagocytes (APCs) being macrophages and dendritic cells (DCs) which are present in the lymph nodes, liver and spleen. Recognition of PAMPS results in the APCs releasing ‘alarm signals’ like ATP (Matzinger, 1994) which are picked up by the resting monocytes in the bone marrow. These cells are then activated and undergo maturation and proliferation and release M1 (tumoricidal) macrophages and activated DCs into the general circulation.

In the meantime, the dying tumour cells in the tumour microenvironment (due to the EDVs releasing cytotoxic drug intracellularly) release ‘find‐me’ signals such as low levels of nucleotides ATP and UTP, fractalkine, lysophosphatidyl choline, or sphingosine 1‐phosphate, which attract APCs to the sites of death within the tissue (Gregory, 2009). The apoptotic cells that expose ‘eat‐me’ signals such as calreticulin, phosphatidyl serine, on the cell surface, promotes specific recognition by the APC and subsequent internalization of the dying cell (Grimsley and Ravichandran, 2003). The apoptotic tumour cell is degraded intracellularly and the released protein antigens are processed and presented on the cell surface via MHC Class I and II molecules. These APCs then migrate to the draining lymph nodes where they present the tumour antigens to CD4+ and CD8+ T cells (Sagnella *et al*., 2020). Once the CD8+ T cells get activated following recognition of tumour‐specific antigens on the APC surface, they home into the tumour microenvironment, recognize the tumour antigens on the live tumour cells and post‐tumour antigen engagement, they secrete perforins and kill those tumour cells (Sagnella *et al*., 2020).

This cascade of events continues to escalate as more APCs are attracted to the new dying tumour cells, engulf them, go to the draining lymph nodes and activate more CD8+ cytotoxic T cells. This cascade then results in tumour antigen‐specific CD8+ effector memory T cells that provide long‐term immunity to the specific cancer (Sagnella *et al*., 2020).

The polar sites of septum formation in Gram −ve and Gram +ve bacteria are thought to be vestigial sites of cell division left over from bacterial evolution and it is possible that the minicell may have been the primordial cell during the path of bacterial evolution. The minicell may therefore be considered to be a very early ancestor of bacteria that existed millions to possibly billions of years ago.

Today, it re‐emerges to offer a way forward in the intractable problem of cancer treatment. These minicells offer solutions required for treatment of late‐stage cancers with little toxicity, both by killing cancer cells and stimulating a robust anti‐tumour immune response, in effect, allowing the patient’s own immune cells to help in the heavy lifting of getting the patient back on his/her feet. No drug or immunotherapy comes near the minicell as a one‐stop‐shop for cancer treatment and for the next 15 years, the bacterial minicell is likely to radically change how cancer is treated.

## Funding Information

No funding information provided.

## Conflict of interest

The authors have no conflict of interest to declare.
